# Signs of deterioration in infants discharged home following congenital heart surgery in the first year of life: a qualitative study

**DOI:** 10.1136/archdischild-2014-308092

**Published:** 2016-01-28

**Authors:** Jenifer Tregay, Katherine L Brown, Sonya Crowe, Catherine Bull, Rachel L Knowles, Liz Smith, Jo Wray

**Affiliations:** 1 Great Ormond Street Hospital NHS Foundation Trust, London, UK; 2 Clinical Operational Research Unit, University College London, London, UK; 3 MRC Centre of Epidemiology for Child Health, UCL Institute of Child Health, London, UK

**Keywords:** Cardiac Surgery, Monitoring, Patient perspective, Qualitative research, Comm Child Health

## Abstract

**Aims:**

To describe the ways in which parents recognise and make decisions about their child's symptoms following discharge home after congenital heart interventions in the first year of life and their experiences of seeking help.

**Methods:**

This was a qualitative study involving semistructured interviews with parents. Twenty-one parents were recruited to the study. Parents all had a child who had congenital heart surgery in their first year of life between September 2009 and October 2013 at one of three UK cardiac centres; the children had either died or were readmitted as an emergency following initial discharge.

**Results:**

Some parents were unable to identify any early warning signs. Others described symptoms of deterioration including changes in feeding and appearance, respiratory distress and subtle behavioural changes that may not be routinely highlighted to parents at discharge. Several barriers to accessing prompt medical assistance were identified including parents feeling that their concerns were not taken seriously, long wait times and lack of protocols at A&E.

**Conclusions:**

Our study highlights behavioural symptoms as being a potentially underemphasised sign of deterioration and identifies a number of barriers to parents accessing support when they are concerned. It is important that parents are encouraged to seek advice at the earliest opportunity and that those health professionals at the front line have access to the information they need in order to respond in an appropriate and timely way. A role for home monitoring was also noted as potentially useful in identifying at risk children who appear clinically well.

**What is already known**

- Congenital heart disease accounts for 3% of infant mortality in the UK, with many children requiring major surgery in the first few days or weeks of life.
- Children with residual complex needs are often discharged to be cared for by their parents at home.
- These children often remain vulnerable to rapid deterioration, and therefore, it is essential that parents are able to identify symptoms of deterioration and access appropriate and timely medical intervention.

**What this study adds**

- Information given to parents is not always sufficient to enable them to make timely decisions about their child's symptoms.
- Behaviour change was noted to be a sign of deterioration that may not be routinely discussed with parents.
- Local health professionals must have sufficient knowledge and information in order to respond quickly and appropriately to parent's concerns.

## Background

UK congenital heart diseases (CHDs) audit data indicate that the number of operations in small infants with more complex CHDs such as hypoplastic left heart syndrome is increasing year-on-year, with more of them surviving the initial operation.^1^ Even after successful cardiac interventions and appropriate hospital recovery, some infants, particularly those with functionally univentricular hearts and those with associated medical conditions,^2^ ^3^ remain fragile when they are discharged home. For example, UK audit data from two cardiac units for the years 2000–2009 found that 11% of neonates died within 30 days of surgery for CHD, but a further 7% died before their first birthday despite apparently successful surgery.^3^ Around half of these deaths occurred in the community or after unexpected emergency readmission. Babies discharged following palliative surgery that are awaiting subsequent staged surgeries can be particularly vulnerable to rapid deterioration, and this makes the role of parents particularly critical in recognising signs of deterioration in their child at an early stage to ensure that they receive appropriate and timely medical intervention.

To understand more about parent perspectives of caring for a child with complex needs after congenital heart surgery, we undertook a qualitative study involving semistructured interviews with parents. Our study aimed to describe the ways in which parents recognise and make decisions about their child's symptoms, their experience of seeking help when there was a concern and any barriers they encountered when seeking help.

## Methods

Parents were invited to take part in the study if their child underwent major congenital heart surgery in their first year of life at one of three UK children's hospitals and subsequently died or were readmitted unexpectedly to intensive care following their initial discharge. Parents were invited to interview if their child had their index surgery in the last five years and were initially approached by specialist nurses who obtained consent to pass their details to the research team.

Interviews were conducted face-to-face by a single researcher (JT) and all but one took place in parents’ own homes. Parents were asked about caring for their child at home following surgery, support they received and events leading up to any emergency readmissions. Interviews were tape-recorded and transcribed verbatim before being analysed using Framework Analysis. Framework Analysis^4^ is a structured approach to managing qualitative data that aims to reduce bias and make analysis of large data sets more manageable. Analysis involves the construction of a series of ‘frameworks’ or grids into which summarised qualitative data are entered under descriptive headings generated by the research team after careful examination of the transcripts. Data from each transcript are entered into the framework, which are then used to extract key themes relevant to the research questions. In the development of frameworks for the present study, each transcript was read by at least three members of the research team.

## Results

### Descriptive information

Specialist nurses contacted 25 families, 21 of whom agreed to be interviewed. One family was excluded as they did not meet inclusion criteria, leaving a total of 20 families who were interviewed for the study. Of these families, 11 were bereaved. Fourteen interviews were conducted with one parent alone (N=14 mothers), and six with both parents together. A range of ethnic, educational and socio-economic backgrounds were represented in the sample (see table 1). One parent did not speak English as a first language and two were bilingual.

**Table 1:** Family demographics

Demographic information	Number
Ethnicity (of child)	
White	
British	14
European	1
Asian	
Bangladeshi	1
Pakistani	1
* *Other	1
Mixed	
* *White/black African	1
* *White/black other	1
Educational history (primary caregiver)	
Learning disability	1
Primary/secondary	11
Graduate	5
Postgraduate	3

All children had their first surgery between September 2009 and October 2013. Following their initial surgery, 12 children were discharged home directly from the specialist surgical centre; the remainder were discharged to their local hospital in a ‘step-down’ arrangement. A case-by-case summary of complications and symptoms for each patient is provided in table 2.

**Table 2:** Case-by-case summary of diagnoses, complications result in readmission and symptoms

Identifier^1^	Diagnosis	Complication	Symptoms
FR01	Single-ventricle disease (not HLHS)	Right diaphragm palsy with plication	Respiratory distress
FR02	HLHS	Wound infection (emergency sternal wound debridement)	Reduced feeding ++vomiting Abdomen ‘not quite right’ (respiratory distress)
FB03†	Transposition of the great arteries (plus or minus other features)	Out-of-hospital cardiac arrest (cause unknown)	Collapsed at home after a feed No other symptoms
FB04†	HLHS	Sudden collapse at home. Died in A&E	Excessive crying and screaming Collapsed at home
FB05†	HLHS	Aspirated at home	Reduced feeding ++vomiting Dry nappy
FB06†	Ventricular septal defect plus significant medical comorbidity	Sudden collapse at home	Slight cough
FR07	Single-ventricle disease (not HLHS)	Blocked left shunt (urgent redo)	Respiratory distress Recessing chest, Purple fingertips and lips Did not notice first time but symptoms pointed out at routine scan at local hospital
FR08	Tetralogy of Fallot	Blocked shunt (urgent central shunt)	No obvious symptoms Low sats detected during home visit
FR09	Transposition of the great arteries (plus or minus other features)	Resection of aortic aneurysm	No obvious symptoms Mother felt something was wrong but did not know what it was ‘mother's instinct’
FB10†	Single-ventricle disease (not HLHS) and significant medical co morbidity	Aspirated at home	Breathing Pale colouring ++vomiting Crying Found in the night in respiratory distress
FB11†	HLHS	Sudden collapse at home. Died in A&E	Excessive crying Unable to settle
FB12†	Single-ventricle disease (not HLHS)	Blocked shunt	Breathlessness noted by paediatrician 2 days before, appeared ‘normal’ to parents Sleepier after feeds Screaming and unable to comfort Collapsed at home
FB13†	HLHS	Blocked shunt	No obvious symptoms Routine vaccinations day before and following this: More lethargic than usual Diarrhoea in night Deteriorated over the course of the day Respiratory distress ->collapsed at home
FB14†	Anomalous coronary artery from pulmonary artery	Sudden collapse at home. Died in A&E	Sweating++ and vomiting after feeds, ‘Spelling’ intermittently (pale/blue/grey lips) Quiet and weak in the AM Grunting/straining sounds Started screaming during feed and collapsed
FR15	Single-ventricle disease (not HLHS)	Severe mitral regurgitation and left ventricular failure	Reduced appetite Lethargic Vomiting Cough ‘Grunting’ breath sounds
FR16†	Single-ventricle disease (not HLHS)	Pacemaker pocket infection and dehydration	++crying and restless at night Rapid breathing Blue face/hands/lips Recessing under ribs
FB17†	Single-ventricle disease (not HLHS)	Sudden collapse at home. Died in A&E	Appeared ‘agitated’ and generally less settled Taking less feed in one go Slight cough Routine vaccinations week prior to death. Collapsed during feed
FB18†	Single ventricle disease (not HLHS)	Sudden collapse at home. Died in A&E	More ‘emotional’ and ‘moody’ More difficult to comfort Easily tired Breathless on activity Napping more during daytime More unsettled at night Blue hands and feet at times Vomiting in hot weather Sweating Woke in night crying, vomiting and short of breath, then screamed and collapsed
FR19	Transposition of the great arteries (plus or minus other features)	Readmitted for coarctation repair	High blood pressure picked up at local review resulting in readmission to tertiary centre No clinical signs observed
FR20	Total anomalous pulmonary venous drainage	Increasing left upper pulmonary vein stenosis, ongoing tachypnoea and hepatomegaly with increased right heart pressures secondary to increased pulmonary venous pressures	General ‘grumpiness’ Mother feeling that something was not right High rate of breathing Vomiting

Broad diagnostic grouping have been used to preserve the anonymity of patients with rare conditions.

†Indicates that the child is deceased.

HLHS, hypoplastic left heart syndrome.

Data are presented in three sections. The first section (Symptoms) describes symptoms of deterioration noted by families, the second section (Decision-making about symptoms) focuses on decision-making about symptoms and the final section (Experience of seeking help) describes families’ experiences of seeking help. Additional quotes are presented in boxes 1–7.

Box 1 **Selected quotations from parents whose child showed no obvious symptoms of deterioration**

“The whole thing of, ‘You will know when your child is so unwell.’ Well, clearly he deals with it pretty well.” (FR09)

“Everything was normal, literally, until the morning that we lost her.” (FB12)

“She was absolutely fine, she was feeding, she was doing everything that we needed her to do, and she was kind of growing nicely. So I think it came as a bit of a shock.” (FR19)

Box 2 **Selected quotations from parents describing feeding and gastrointestinal symptoms in their child**

“The last week of her life, she started being sick and she's never been sick. And I said to the community nurse “she's been a bit sick and she's not bringing up her wind…There's something wrong.” (FB05)

“He was sick the whole time. He couldn't drink a bottle without being sick.” (FR09)

“I think it was around that time he started to take off his feeds as well.” (FB17)

“One thing we did notice was that if it was quite a hot day, he would be a bit sick.” (FB18)

Box 3 **Selected quotations from parents descriptions of respiratory distress in their child**

“It was his tummy and he was just, it wasn't he was breathing funny, but it was just something-, he wasn't quite comfortable. Something wasn't quite right.” (FB02)

“That little bit of the whistle in her chest again and basically just the breathing, you know, with the ribs. I don't know how to describe it. The tummy sort of goes right in and then you can see, like, the outline of the ribs when someone's breathing a bit funny.” (FR07)

“In the morning, when he'd wake up, he'd cough. I now learned that that's because he had lots of fluid resting on his lungs…..At the end of August he started wheezing, but really randomly, to the point that I knew something was wrong.” (FR09)

“We woke up in the night time, and we noticed that [his] breathing-, he looked different and his breathing was very hard.” (FB10)

“‘Is she more breathless?’ I don't actually know whether she was or not….I think it's quite hard if it's a gradual thing. To this day I still don't really know whether she was more breathless or not, but she was her normal usual self.” (FB12)

“Sometimes she would do a noise, as if she was straining.” (FB14)

“His nose will flare.” (FR16)

“I noticed around seven months he would be a bit more breathless, he would have to nap more often because he would get tired more often….His stomach was sinking in.” (FB18)

“He was breathing quite fast and I was like, ‘Slow down, slow down’, I didn't know breaths could get that fast.” (FR20)

Box 4 **Selected quotations from parents describing changes in their child's appearance**

 “We, kind of noticed her fingernails at the end were going a little bit purple. Just around here, of her lips, they were a little bit purple.” (FB02)

“He was smiling and happy but when he was lying down he just looked a bit-, in hindsight, he was incredibly puffy.” (FR09)

“Sometimes he was a bit pale.” (FB10)

“I noticed that she was sweating in her hair…When I stripped her off, then I noticed her chest was blue, and that-, I would have said she was sweating, but actually in retrospect she was clammy.” (FB12)

“Her colour is a dark grey, blue and yellow. You see? There is no colour in her lips…..My husband couldn't tell….nobody could tell, except me.” (FB14)

“I found that he would, even if it was very cool…in his sleep, he would sweat an awful lot. Around his back, his shoulders, his neck. I started to notice the blueing of the hands and his mouth….I think from six months on I mentioned at every appointment that I would notice more often that his hands would get blue, his feet would get blue, his lips.” (FB18)

“He'll go pale in his face, his hands will go cold and his lips will turn blue.” (FR21)

Box 5 **Selected quotations from parents describing a range of behavioural changes in their child**

“It was quite sudden for us. He cried quite a lot and we could not know that he is not well. There was not any sign that he was not well.” (FB10)

“Apart from the day he passed away, that was the only time [we noticed anything]. Then I wasn't overly concerned, to me he was just crying, he didn't look any different. [We] just couldn't stop him crying…Probably for a normal child, baby, that's normal. I don't know, being a new mother, whether that's normal or not normal. He was crying, and after everything I'd tried, he still wouldn't stop crying.” (FB11)

“I did wonder if she was head bobbing a little bit, but thought ‘She's really tired,’…so I took her back up with me and literally within five minutes of putting her down she just started screaming.” (FB12)

“She wasn't herself, she was a lot quieter than she usually was, a lot drowsier.” (FB13)

“He'll start crying a lot more, he'll wake himself up…..He was really unsettled, he wouldn't sleep……[later:] He kept hurling himself over, screaming like he was in pain.”(FR16)

“He would have to nap more often…Also he started to get more unsettled at night. He was just more unsettled generally. “ (FB18)

“I felt something was wrong, but I couldn't put my finger on it.” (FR20)

Box 6 **Selected quotations illustrating parent's decision-making about their child's symptoms**

“We just said [baby] just needs to be known, that's all it is, just get known because if you present him in an iller condition, he's deteriorating, they need a baseline to compare it against.” (FR01)

“It felt like total care to none at all.” (FB11)

“I think that was the problem in the end because I was so focussed on the paperwork than actually my baby. I couldn't see what else was going on with her because I was so worried about every drop of milk.” (FB05)

“That's the first time I'd seen anything like that, so I wasn't really aware of what was going on until they said, “well okay, this is the kind of stuff you need to look for…I think once you've seen it once you can tell after that.” (FR07)

“When I really feel strongly about something I just have to act on it and I need to take him to see someone and then I can go, ‘Look, I just think that there's something wrong here. Help me out, because I can't tell you what it is.” (FR09)

“They told you what to look out for, his blue lips and his eyes, but I think from a parent point of view, you do not really see it as much as a medic would.” (FB11)

“I think it's quite hard if it's a gradual thing. To this day I still don't really know whether she was more breathless or not, but she was her normal usual self.” (FB12)

Box 7 **Selected quotations illustrating parent's descriptions of seeking help when they were concerned about their child**

“[Our cardiologist] gave him some antibiotics and it cleared up, but he said ‘At what point were you going to go to your GP?’ We said, ‘We see you.’…there's a threshold—we went the wrong side of it and [the cardiologist] reigned us back in.” (FR01)

“I feel like my community, my borough itself was the biggest let down for me, the hospital being the first one.” (FB04)

“They need to listen, ‘cause that nurse that came here, she didn't listen to me that day….and she was saying ‘Wait until the meeting next week’ [review appointment at the specialist centre]…They're drumming it into me ‘make sure this, make sure that’…and then they let her down.” (FB05)

“I have always questioned, ‘Should I contact her?’…'Maybe I'm just being a little paranoid,’ but who cares? She's never ever gone, ‘I really wouldn't worry about it’. (FR09)

“What gets me is that, I have all the information, as in I know all the symptoms, I know the nitty gritty, and I feel, sometimes, that I'm not listened to very well.” (FR15)

“They had originally told me that being his mum….I would know the signs…then when I started to notice things…and I told them, I just felt like I wasn't being listened to.” (FB18)

“It didn't seem like there was any plan in place at all. They didn't know how to deal with it or what to do…They were actually trying to look through [his] red health record book for answers…” (FR19)

“If he is sick, I have to decide by 5 o'clock how sick I think he is. Even in A&E then, there is nobody around except some overworked registrar who might not know him so well and not be so experienced, so it has got to be done in the day or not at all……You have to be pushy, you have to be proactive, otherwise I don't know what would happen.” (FR20)

### Symptoms

#### No symptoms

A small number of families reported very mild or no obvious symptoms at all followed by the very sudden collapse and deterioration of their child. Two apparently non-symptomatic children were readmitted as a result of routine saturation and blood pressure monitoring, which identified a blocked shunt in one case and the imminent need for further palliative surgery in another.

#### Feeding and gastrointestinal symptoms

Many families noted changes in their child's feeding behaviour, which included reduced feeding, increased lethargy during feeds and the presence or increase in vomiting. For many families, these symptoms came on gradually and presented in the context of challenging feeding behaviour characteristic of cardiac babies. In one case, a prolonged bout of diarrhoea following routine vaccinations the previous day resulted in the very rapid decline of a previously well child.

#### Respiratory distress

A variety of terms were used by parents to describe respiratory symptoms in their baby. These included descriptions of breath sounds such as ‘wheezing’, ‘grunting’, ‘straining’ and ‘whistle’; changes in the rate, pattern or work of breathing including ‘breathlessness’; or their child's appearance such as flaring nostrils and recessing under the ribs, which was sometimes described by parents as an abdominal symptom.

#### Appearance

It was common for parents to describe changes in their child's appearance. This included changes in colour around the lips and extremities, which in the earlier stages may have been transitory, appearing in ‘spells’ or during exertion. Interestingly, there was variation in the ways that parents described the colour of their child: ‘blue’, ‘purple’, ‘pale’, ‘grey’ and ‘yellow’. Some parents reported that the colour changes were so subtle that they either missed them completely or they were not apparent to anyone but them. Colour change sometimes presented in conjunction with cold extremities. Some parents also reported that their child had started to become more ‘sweaty’ or ‘clammy’, particularly at night or during a feed.

#### Behaviour

Many parents described behaviour changes in their child. These were typically subtle and not dissimilar to behaviours exhibited by healthy babies, which made them particularly difficult for parents to interpret. Sometimes these subtle behavioural changes were the sole indicator that the child was unwell. In the early stages of their child's deterioration, several parents noticed their child becoming increasingly weak and lethargic and tiring more quickly during exertion or feeds. Some parents noted changes to their child's sleep pattern with them sleeping more during the day and waking more frequently in the night.

Another early sign was their child being generally more ‘moody’, ‘grouchy’, ‘emotional’, ‘agitated’ or ‘unsettled’, and generally ‘not themselves’. Parents described babies that cried more frequently and were more difficult to comfort than usual. The changes they described were not out of keeping with what would be expected for a healthy baby but they were unusual for their own child. Several parents found this very difficult to interpret and described a feeling of knowing something was wrong but being unable to identify what it was. In the later stages, a number of parents reported these behavioural symptoms increasing dramatically into persistent crying followed by high-pitched ‘screaming’ that preceded their child's rapid deterioration.

### Decision-making about symptoms

While many, but not all, parents recalled being given information about signs and symptoms during their child's hospital admission, this was not always sufficient to enable them to recognise these symptoms out in the community. Even when symptoms were recognised, parents sometimes struggled to describe these and to make decisions about a course of action. This was particularly true if symptoms appeared very subtle or had a gradual onset. One parent commented that as this was her first child she found it difficult to evaluate symptoms as she did not know what was ‘normal’ for a healthy baby. Several parents spoke of the burden of completing monitoring forms at home, particularly in relation to feeding, with one parent explicitly stating that she felt she may have missed early warning signs in her child as a result. There was also some difficulty identifying change when the child's baseline was atypical. A small number of parents said that they did not recognise the symptoms on the first occasion, but that once these had been pointed out to them on their own child, they found it much easier to identify them on subsequent occasions.

Often decision-making about symptoms took place in the context of local services that were relatively unfamiliar with CHD and, in some cases, this had a detrimental effect on parent's trust in their local hospital. Despite this, some families still recognised the need for their child to be known to local services.

### Experience of seeking help

For symptoms that parents judged to be non-urgent, their first point of contact would typically be the health professional with whom they had the best relationship—often the cardiac liaison nurse at their specialist centre or the community nurse. However, in some cases, parents waited until their next follow-up appointment to discuss their concerns with their local paediatrician or their cardiologist, resulting in a delay in their child receiving treatment. Several parents mentioned fear of appearing ‘silly’ or ‘paranoid’, particularly to more senior health professionals, although this could be countered by positive experiences of seeking help and reassurances at an early stage—typically from the liaison nurse—that they should phone with any concern no matter how small.

Parents reported an overwhelmingly positive experience of the support they received from their cardiac liaison nurse with this link being described by one family as a ‘lifeline’. This was particularly true if this was someone they had met during their hospital admission. In some cases, the liaison nurse was able to liaise with a family's local hospital to facilitate more rapid access and treatment in an emergency, discuss treatment plans with local health professionals and arrange transport back to the specialist centre when required.

Not all families had a good experience of seeking help and several families felt their concerns were not taken seriously by local services. In some cases, parents were falsely reassured about symptoms or told to wait until their next follow-up appointment to discuss their concerns with their cardiologist. Several parents described occasions when they had to be particularly assertive with health professionals in order to get the right care for their baby.

Several parents felt that their local A&E was unprepared to manage a child with CHD in an emergency and described staff having difficulty accessing the information they needed to treat the child. Out-of-hours service at A&E was also raised by some parents, with many detailing long wait times and one parent describing having to make decisions about the severity of her child's symptoms within working hours in order to get the best care.

## Discussion

Our study details parent accounts of making decisions about their child's symptoms at home after congenital heart interventions in the first year of life and their experiences of seeking help. Our findings suggest that, while the potential for postdischarge deterioration in these children is well known,^2^ ^3^ information given to parents is not always sufficient for them to make decisions about their child's symptoms and summon appropriate support. One difficulty is in the language used to describe symptoms to parents. For example, some classic descriptions of heart failure describe ‘blue’ skin colour, which several parents in our study found ambiguous and difficult to interpret. Decision-making may be particularly challenging if symptoms appear gradually or if parents have no previous experience of seeing their child unwell. Behavioural symptoms, such as changes in sleep pattern, lethargy, crying and irritability, were also highlighted as a potentially under-recognised sign of deterioration in these children. These symptoms were difficult to interpret as they often presented subtly at first and could be difficult to distinguish from behaviour typical of a healthy infant. It is important that parents are encouraged to seek advice at the earliest opportunity and that health professionals at the frontline have access to the information they need to respond in an appropriate and timely way. We suggest that such subtle signs as a behaviour change should be considered within the wider context of an infant's medial history, ‘normal’ clinical state and physical examination at a given time point, since these may represent an early warning of true deterioration.

While it is important for parents to be trained to recognise symptoms of deterioration in their child, it is also important that they are able to summon prompt and appropriate medical care when they have a concern. Barriers to accessing such assistance included parents’ fear of appearing ‘silly’ or ‘paranoid’, feeling their concerns were not taken seriously, and long wait times and lack of protocols at A&E. Several parents described feeling let down by their local services after flagging symptoms of concern and either being falsely reassured or advised to wait until their next follow-up to speak with their child's cardiologist. Protective factors included parents having a trusted point of contact with whom they could safely discuss their concerns, and having the confidence to assert themselves with health professionals when they were not satisfied that these had been addressed. A role for home monitoring^5–7^ was also noted as potentially useful in identifying high-risk children. In some cases, apparently asymptomatic children were identified with the aid of measurements taken at routine follow-up, suggesting that these more objective forms of surveillance may be effective for identifying children who require intervention but appear clinically well.

Our study has a number of limitations. First, parents approached to take part in the study were those known to specialist nurses who assisted with recruitment. While this meant that parents were approached by someone familiar to them resulting in a high opt-in rate, it is possible that families opting into the study were those who had a better relationship with the nurses at their hospital. An important consideration of qualitative research methods is to describe, rather than quantify, the views held by a population of interest; therefore, it is important to ensure that the study sample represents the diversity present in the population being described. Our study included parents of children with a range of diagnoses, outcomes and discharge pathways. We also attempted to achieve diversity in our sampling of ethnicity and parent educational level, although, as is often typical in UK research, our sample included parents of predominantly white British children. Our recruitment strategy was also not optimised to recruit parents whose first language was not English, despite offering access to interpretation, and it is likely that these parents face additional challenges not captured by this study.

Many complications that arise following congenital heart surgery can lead to relatively rapid deterioration in a small infant leaving a small window of opportunity to intervene. This makes it particularly important for parents to be supported to recognise symptoms in their child and for them to be able to summon help quickly when there is a concern. The family burden of caring for a child with complex health needs is well known,^8–14^ and since families responding to their infant's symptoms are likely to be acting under stress, it is important that the information they are provided with is straightforward and that help is readily accessible. Our study has implications for health professionals involved in the discharge and follow-up of babies after congenital heart surgery, both in relation to interpretation of reported symptoms and the processes they follow in response.
